# Reply on ‘High-urgency heart transplantation and outcome trade-offs: early post-transplant infection and mortality’

**DOI:** 10.1093/eschf/xvag186

**Published:** 2026-06-27

**Authors:** Hyun-Jai Cho

**Affiliations:** Department of Internal Medicine, Seoul National University Hospital and Seoul National University College of Medicine, Seoul, Republic of Korea

**Keywords:** Heart transplantation, Urgency Status, Post-transplant infections, Mortality, Korean Organ Transplant Registry

We thank Biswas and colleagues for their careful reading of our study and for raising two thoughtful methodological points. We welcome the opportunity to clarify.

Time-varying immunosuppression.

We agree that the balance of immunosuppression is central to post-transplant infection, which is precisely why we dedicated a full results section, Table 3, and Supplementary Figure S9 to it—including the steroid-tapering × urgency interaction (*P* for interaction = .008) that the correspondents cite, which was in fact reported by us. We respectfully disagree, however, that this variable should have entered the primary early-infection models in Supplementary Table S4. Post-transplant immunosuppression is adjusted dynamically in response to the evolving clinical course—renal dysfunction limiting tacrolimus, corticosteroid dependence in sicker recipients, and infection itself. It therefore lies on the causal pathway between urgency status and infection rather than acting as an independent confounder, and conditioning on it would introduce mediator and collider bias that attenuates, rather than clarifies, the urgency effect. Temporality reinforces this: the dominant signal is 1-month infection, which largely precedes the steroid divergence observed at 6 months and 1 year. Notably, during peer review we were explicitly cautioned against causal interpretation of steroid exposure and accordingly reframed the higher steroid doses in Status 0 recipients as a reflection of underlying clinical complexity rather than a cause of adverse outcomes. Promoting time-varying immunosuppression to a ‘corrective’ covariate would run counter to that deliberately cautious interpretation. We agree the differential steroid-infection association is hypothesis-generating and merits dedicated prospective evaluation.

Donor characteristics.

This concern is partly addressed in the original report. Procurement- and donor-related variables that were captured—cold and warm ischaemic time, panel-reactive antibody >50%, and donor-specific antibodies—are presented in Table 1, and cold ischaemic time was included as a covariate in the multivariable mortality models. Importantly, ischaemic times and donor-specific antibody positivity did not differ between urgency groups, making differential donor ischaemic injury or allosensitization an unlikely explanation for the urgency-related infection gap. We acknowledge, as stated in our limitations, that granular donor variables (donor age, cause of death, and donor infectious serology) were not uniformly available in the KOTRY heart cohort during the study period, and we agree this is a genuine registry constraint. We would note, however, that donor-derived infections constitute a minority of early post-transplant infections, and the pattern we observed—early, predominantly bacterial, respiratory-tract infection tightly linked to prolonged mechanical ventilation—points to recipient critical illness rather than donor transmission. Consistent with this, the outcomes most sensitive to donor quality and immunological mismatch, namely acute rejection and cardiac allograft vasculopathy, did not differ between urgency groups, arguing against donor quality as the principal driver of the differential infection burden.

We thank the correspondents again for their constructive engagement and agree that future linkage of recipient registries to detailed donor datasets, together with time-varying immunosuppression modelling designed to avoid over-adjustment, would further strengthen causal inference in this high-risk population.

## Declarations

### Data Availability

No data were generated or analysed for this manuscript.

